# Analyses of phenotypic differentiations among South Georgian Diving Petrel (*Pelecanoides georgicus*) populations reveal an undescribed and highly endangered species from New Zealand

**DOI:** 10.1371/journal.pone.0197766

**Published:** 2018-06-27

**Authors:** Johannes H. Fischer, Igor Debski, Colin M. Miskelly, Charles A. Bost, Aymeric Fromant, Alan J. D. Tennyson, Jake Tessler, Rosalind Cole, Johanna H. Hiscock, Graeme A. Taylor, Heiko U. Wittmer

**Affiliations:** 1 School of Biological Sciences, Victoria University of Wellington, Wellington, New Zealand; 2 Aquatic Unit, Department of Conservation, Wellington, New Zealand; 3 Museum of New Zealand Te Papa Tongarewa, Wellington, New Zealand; 4 Centre d’Etudes Biologiques de Chizé, CNRS-Université de la Rochelle, Villiers en Bois, France; 5 Murikihu District Office, Department of Conservation, Invercargill, New Zealand; Centre National de la Recherche Scientifique, FRANCE

## Abstract

Unresolved taxonomy of threatened species is problematic for conservation as the field relies on species being distinct taxonomic units. Differences in breeding habitat and results from a preliminary molecular analysis indicated that the New Zealand population of the South Georgian Diving Petrel (*Pelecanoides georgicus*) was a distinct, yet undescribed, species. We measured 11 biometric characters and scored eight plumage characters in 143 live birds and 64 study skins originating from most populations of *P*. *georgicus*, to assess their taxonomic relationships. We analysed differences with principal component analyses (PCA), factorial ANOVAs, and Kruskal-Wallis rank sum tests. Results show that individuals from New Zealand differ significantly from *P*. *georgicus* from all other populations as following: 1) longer wings, 2) longer outer tail feathers, 3) deeper bills, 4) longer heads, 5) longer tarsi, 6) limited collar extent, 7) greater extent of contrasting scapulars, 8) larger contrasting markings on the secondaries, 9) paler ear coverts, 10) paler collars, and 11) paler flanks. Furthermore, we used a species delimitation test with quantitative phenotypic criteria; results reveal that the New Zealand population of *P*. *georgicus* indeed merits species status. We hereby name this new species *Pelecanoides whenuahouensis sp*. *nov*. Due to severe reductions in its range and the very low number of remaining birds (~150 individuals limited to a single breeding colony on Codfish Island/*Whenua Hou*) the species warrants listing as ‘Critically Endangered’. An abstract in the Māori language/*Te Reo Māori* can be found in S1 File.

## Introduction

Conservation biology remains focussed on species as distinct and single ecological and taxonomic units and accurate nomenclature and taxonomic placement of threatened species is thus crucial to effective conservation management [1, 2]. If common, polytypic taxa (i.e., clusters of distinct and diverged species [3]), encompass unclassified taxonomic units warranting species status, their biodiversity value remains “hidden”. Valuable time to implement conservation management may consequently be lost if composite “species” ameliorate the actual conservation status of threatened taxa [2]. Therefore, the “hidden” rare taxa are unlikely to receive the management required to conserve them.

This phenomenon of “hidden” but endangered taxa is common on archipelagos and many distinct and endemic taxa on isolated islands are consequently underappreciated [2]. For instance, the taxonomy of many species complexes on the archipelago of New Zealand remains unresolved e.g., [1, 4, 5]. New Zealand’s diverse seabird community, containing some of the most threatened seabird species in the world [6, 7], remains in taxonomic flux (e.g., [8–13]) and several undescribed and threatened seabird taxa may remain within polytypic seabird “species” in New Zealand.

The taxonomy of Diving Petrels (*Pelecanoides* spp.) is confusing, as all four currently recognized species (*P*. *garnottii* [14], *P*. *magellani* [15], *P*. *georgicus* [16], and *P*. *urinatrix* [17]) are cryptic, extremely similar, and restricted to remote offshore islands [18–22]. Given these identification challenges, Murphy and Harper [23] hypothesised the potential presence of undescribed taxa on less well-studied Subantarctic islands. The South Georgian Diving Petrel (*Pelecanoides georgicus* [16]) may be a potentially polytypic seabird taxon that is currently considered monotypic [18], common (15 million individuals), and widespread (occurring throughout the southern oceans [21]). Therefore, this species is listed as ‘Least Concern’ by the IUCN [24], but it may comprise several distinct and highly threatened taxa [23, 25, 26]. South Georgian Diving Petrels occur across the southern hemisphere with remaining, allopatric colonies on South Georgia (U.K.), Prince Edward Island (South Africa), Crozet Islands (France), Kerguelen Islands (France), Heard Island (Australia), Macquarie Island (Australia), Bishop Islet (Australia), and Codfish Island/*Whenua Hou* (New Zealand) [19, 27, 28]. Other colonies were extirpated by introduced species including colonies on Marion Island (South Africa) and southern New Zealand (Enderby and Dundas Island on the Auckland Islands, Chatham Islands, Stewart Island, and the South Island) [6, 19, 29, 30].

The Macquarie Island population of *P*. *georgicus* was assigned to a subspecies (*P*. *g*. *novus*) [25], but this taxon is considered a junior synonym to *P*. *georgicus* and thus not adopted in current taxonomy [18, 31, 32]. However, its taxonomic status has never been formally re-examined. The population on Macquarie Island was considered extinct [19, 33, 34], but the species appears to be slowly recolonizing the island [27] and a relict population has been discovered 33 km south of Macquarie Island on Bishop Islet [28, 35]. The status of *P*. *georgicus* on Macquarie Island (three to four pairs) is precarious, yet the other Australian population on Heard Island numbers 10,000–100,000 individuals and so the overall *P*. *georgicus* Australian population is listed as ‘Vulnerable’ [35].

In New Zealand, the *P*. *georgicus* population may be a distinct taxon, as highlighted by ecological, molecular, osteological, and parasitological data. Specifically, *P*. *georgicus* in New Zealand prefers sandy foredunes at sea level for breeding [6, 36], rather than scree at higher altitudes as individuals from other populations do [19, 28, 37, 38]. Results of a preliminary molecular analysis, using the mitochondrial (12S) ribosomal RNA gene, suggest that the New Zealand population diverged from *P*. *georgicus* populations in the southern Indian Ocean several 100,000 years ago [26]. Furthermore, osteological analyses revealed differences between *P*. *georgicus* populations [39]. Specifically, *P*. *georgicus* bones from New Zealand were 2.5–5.9% larger than bones from Heard Island. Finally, *P*. *georgicus* in New Zealand hosts different feather lice (*Pelmatocerandra setosa*, a species commonly found on *P*. *urinatrix*) than other *P*. *georgicus* populations (*Pelmatocerandra enderleini* [40]). Nevertheless, the debate surrounding the taxonomic status of *P*. *georgicus* in New Zealand has resulted in confusion in the literature. For example, Scofield and Stephenson [41] considered the population to pertain to *P*. *urinatrix exsul* [42], but provided no argumentation for this, while differences between *P*. *georgicus* and *P*. *urinatrix exsul* are well known [19, 23, 30, 37, 38] and apply to the New Zealand population [43]. Such confusion is concerning, as the New Zealand population is highly threatened [36, 44], small (approximately 150 individuals; [29], restricted to Codfish Island [30, 36, 44], and thus classified as ‘Nationally Critical’ in New Zealand [45].

In order to resolve the taxonomic status of the threatened relict populations of *P*. *georgicus* on Codfish Island and Macquarie Island, we assessed differences in 11 biometric and eight plumage characters from a total of 207 individuals sampled across their range. We addressed species limits within *P*. *georgicus* using a species delimitation test based on quantitative phenotypic criteria [46], allowing us to reveal a new, distinct species of Diving Petrel.

## Material and methods

### Origin of samples

**Fig 1:**
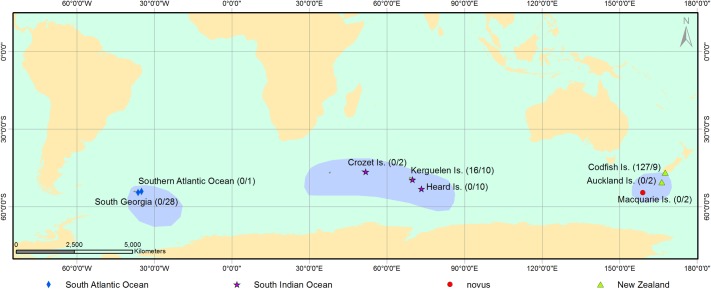
Distribution of *Pelecanoides georgicus* samples. Origin, number, type (live/study skin), and corresponding pool of samples used in the assessment of phenotypic differentiations between allopatric populations of *Pelecanoides georgicus*. The species’ distribution is based on [47].

We assessed biometric and plumage differences between *P*. *georgicus* populations using 143 live adults and 64 adult study skins, covering almost the entire range of the species [19, 47] (Fig 1). JHF measured and ID photographed live individuals on Codfish Island, New Zealand (-46.77, 167.65) (*n* = 127) between 2015 and 2017. CMM, CAB, and AF measured live birds from the Kerguelen Islands (-49.48, 70.05) (*n* = 16) in 2016. In addition, we measured and photographed study skins deposited in Te Papa Tongarewa Museum of New Zealand, Wellington, New Zealand (NMNZ), the American Museum of Natural History, New York, U.S.A. (AMNH), and the Museo Argentino de Ciencias Naturales Bernardino Rivadavia, Buenos Aires, Argentina (MACN). JHF measured and photographed NMNZ study skins originating from: South Georgia (-54.48, -36.28) (*n* = 3), Southern Atlantic Ocean (approx. -54.00, -54.00) (*n* = 1), Crozet Islands (-46.41, 51.80) (*n* = 2), Kerguelen Islands (*n* = 6), Heard Island (-53.08, 73.45) (*n* = 10), Codfish Island (*n* = 9), and Dundas Island, Auckland Islands (-50.58, 166.32) (*n* = 1). JT measured and photographed AMNH study skins originating from South Georgia (*n* = 16), Kerguelen (*n* = 4), and Macquarie Island (-54.77, 158.83) (*n* = 2). AJDT measured and photographed MACN study skins originating from South Georgia (*n* = 7). Furthermore, we requested further data and photographs from the British Museum of Natural History, Tring, U.K. (BMNH). BMNH study skins originated from South Georgia (*n* = 2) and Enderby Island, Auckland Islands (-50.50, 166.30) (*n* = 1). While the identification of Diving Petrels can be confusing, we identified all 207 samples confidently as *P*. *georgicus* (based on white inner vanes of outer primaries, medial position of the paraseptal process, and convergent bill sides [23, 38], including two study skins at BMNH originating from South Georgia (1938.12.19.102 and 1940.12.7.45) that were originally labelled as Common Diving Petrel (*P*. *urinatrix exsul/coppingeri*).

### Biometric characters

We compared 11 biometric variables of individuals from different populations. Nine different biometric variables were measured once (Table 1). Measurements were rounded to the nearest mm for wing and tail measurements and to the nearest 0.1 mm for all other measurements. Where applicable, measurements were taken on the right side of the bird. We used the difference between T1 and T6 to enable quantitative assessment of tail fork depth. Furthermore, we estimated the placement of the anterior end of the paraseptal process in relation to the skull in percentages (posterior = 0%; anterior = 100%) [23]. JHF made 77% of all measurements, while all other measurements were taken by experienced professionals (i.e., curators of natural history museums), except one (JT), who was trained accordingly by JHF. To ensure consistency among measurers, a short video illustrating the precise measuring methodology was also provided to all measurers (S2 File). As such, we assumed to have eliminated measurer bias.

**Table 1:** Definitions and measuring tools for biometric variables of *Pelecanoides georgicus* populations.

Biometric variable	Measuring tool	Definition
Wing length	Wing ruler	Flattened wing chord from carpal joint to longest primary (P10).
Length of T6	Tail ruler	Distance from point of insertion to tip of the outermost tail feather (T6).
Length of T1	Tail ruler	Distance from point of insertion to tip of the innermost tail feather (T1).
Bill length	Dial/vernier callipers	Distance on a horizontal plane from front curve of upper mandible to distalmost crown feathers.
Bill width	Dial/vernier callipers	Width at distalmost crown feathers.
Bill depth	Dial/vernier callipers	Depth (height) of both mandibles at the distalmost crown feathers, including nostrils (nasal tubes).
Arch length	Dial/vernier callipers	Distance from the apex of the lower mandible rami to the distalmost throat feathers.
Head length	Dial/vernier callipers	Distance from the front curve of upper mandible to the supraoccipital.
Tarsus length	Dial/vernier callipers	Distance from the notch between the digits and the tarsometatarsus to the notch between the tarsometatarsus and the tibiotarsus.

### Plumage characters

**Fig 2:**
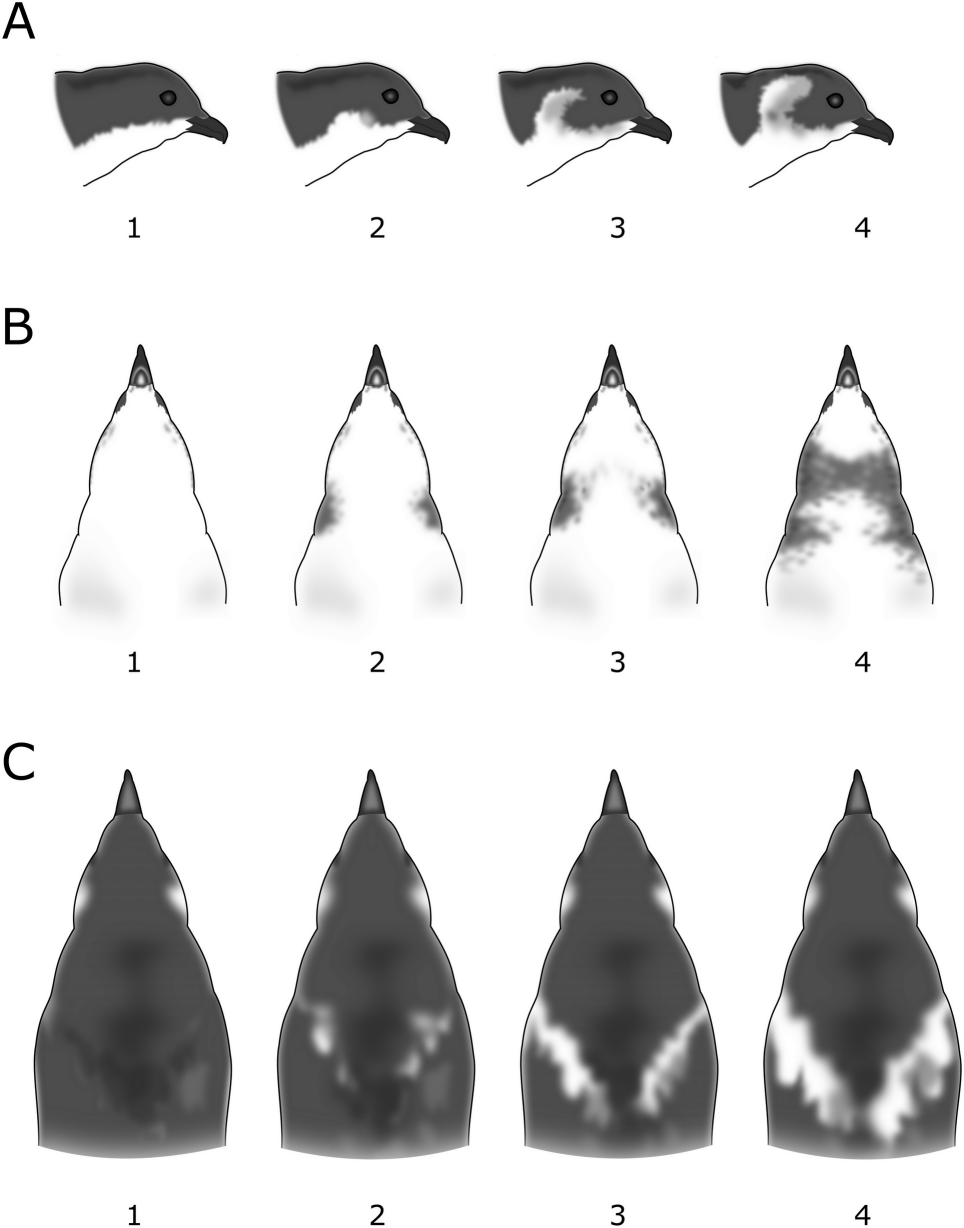
Scoring scale for plumage characters in *Pelecanoides georgicus* populations. (A) Extent of contrasting ear coverts: 1 = absent, 4 = reaching over the eye. (B) Extent of collar: 1 = absent, 4 = fully connected. (C) Extent of contrasting scapulars: 1 = absent, 4 = prominent and virtually connected.

**Fig 3:**
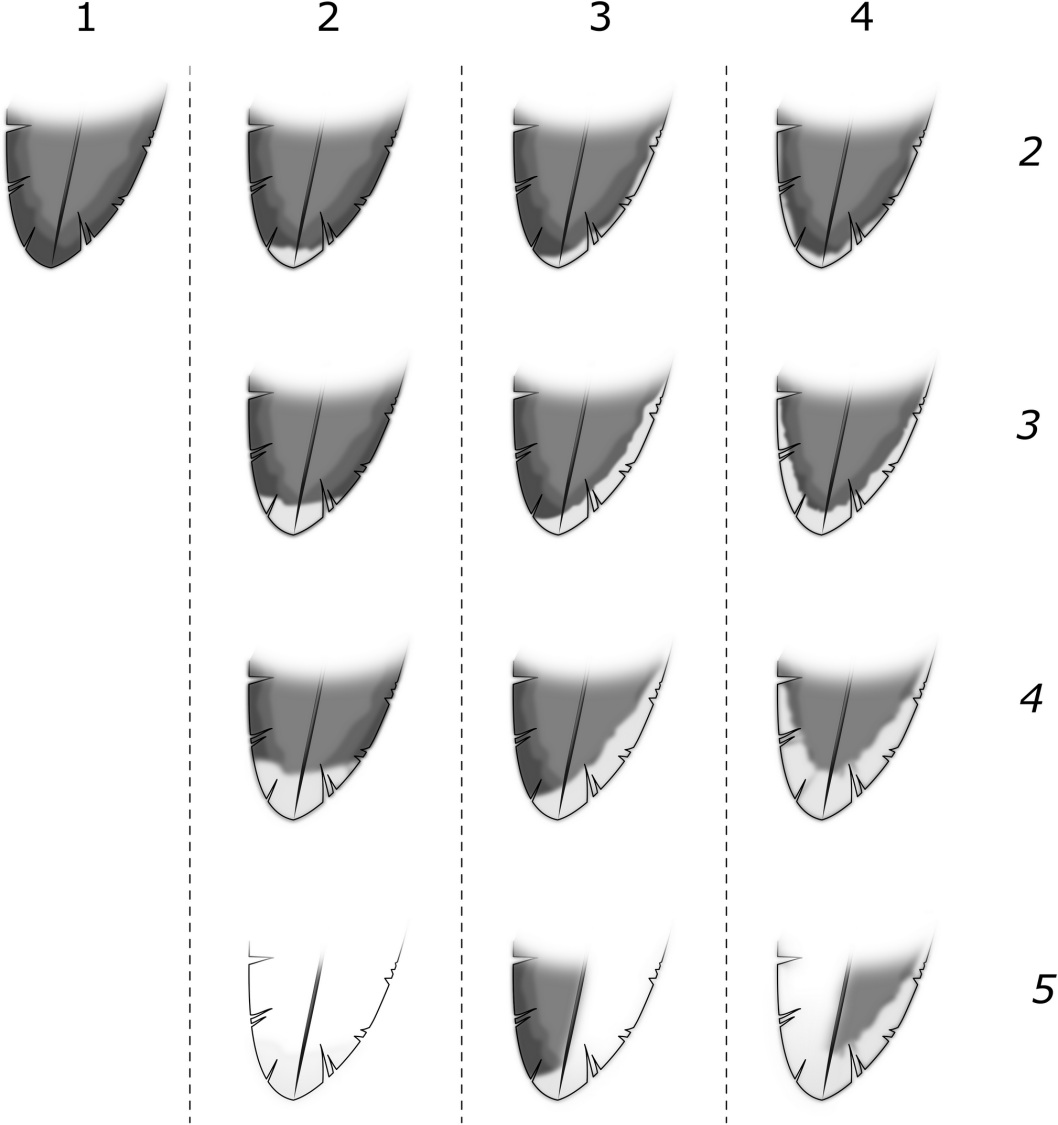
Scoring matrix for shape and extent of variation of markings on secondaries in *Pelecanoides georgicus* populations. Shape (horizontal): 1 = absent, 4 = present on tip, inner and outer vane. Extent (vertical): 1 = absent, 5 = covering at least one vane entirely.

We created a semi-standardised photo archive of live *P*. *georgicus* and study skins and assessed five ordinal plumage characters: contrasting ear-covert extent (1–4; Fig 2A), collar extent (1–4; Fig 2B), contrasting white scapular extent (1–4; Fig 2C), shape of white markings on all secondaries (S1-S10) (1–4; Fig 3) and extent of white markings on all secondaries (S1-S10) (1–5; Fig 3). In addition, we recorded the colour of the contrasting ear coverts, collar, and flank on a scale (1–5; 1 = white, 5 = black). We refrained from using a colour chart, as this tool is not ideal when colours fade into each other [48]. In several taxonomic studies plumage characters are scored on larger scales (e.g., 1–10; [49, 50]). We refrained from using such scales as they come with the arguable difficulty of distinguishing between neighbouring classes.

### Data analysis

We grouped samples of *P*. *georgicus* into four pools: (1) the South Atlantic Ocean population (SAO; *n* = 29), (2) the South Indian Ocean population (SIO; *n* = 38), (3) the New Zealand population (NZ; *n* = 138), and (4) the Macquarie Island population *P*. *g*. *novus* (referred to as novus [25]; *n* = 2) (Fig 1). We accounted for potential differences between live birds and study skins (e.g., through shrinkage or fading; [51–53] by using Welch’s two-sample t-tests with the NZ pool as a subset (as this pool had the largest and most complete data set). Results indicated that measurements of T1 length (*t* = 2.453, *df* = 9.801, *P* = 0.035) and bill depth (*t* = 4.659, *df* = 10.08, *P* < 0.001) showed signs of shrinkage. Additionally, the contrasting ear-covert extent (*t* = 2.695, *df* = 8.500, *P* = 0.026) and flank colour (*t* = 3.537, *df* = 10.706, *P* = 0.04), showed signs of fading. All other biometric measurements and plumage scores did not show signs of shrinkage or fading (i.e., *P* > 0.05). Consequently, we excluded the measurements and scores showing shrinkage/fading from live birds (we only had data from live birds from Codfish Island and the Kerguelen, while we had data from study skins from all sites). As phenotypic differences between sexes have not been recorded [23] and few samples were sexed, potential sexual dimorphism was not taken into account.

We then assessed differences in biometric measurements using a three-step approach: 1) We applied multivariate statistics to assess clustering of pools using two principal component analyses (PCA); one for biometric and one for plumage characters. We replaced missing values with the means of each pool, excluded negative values (tail fork depth), and normalized data before executing the PCAs [54]. We tested for differences between pools within the PCA space with factorial ANOVAs, but excluded pools with small sample sizes (*n* < 7). 2) We then applied univariate statistics to test for significant differences between pools using factorial ANOVAs followed by Tukey HSD tests for biometric characters (e.g., [48]) and Kruskal-Wallis rank sum tests followed by pair-wise comparisons with Welch’s two-sample t-tests for plumage characters (e.g., [49, 50]). We excluded small sample sizes (*n* < 7) from univariate statistics as well. 3) We then addressed the potential species status of the pools with larger sample sizes (*n* < 7) using the species delimitation test with quantitative phenotypic criteria as described by Tobias *et al*. [46].

Following Tobias *et al*. [46], we scored and summed two biometric characters, three plumage characters, and one behavioural/ecological character to address species limits among *P*. *georgicus* populations. Characters with continuous data (i.e., biometric data) were scored based on Cohen’s *d* effect sizes (*d* = 0.2–2.0 = score of 1, *d* = 2.0–5.0 = score of 2, *d* = 5.0–10.0 = score of 3, *d* > 10.0 = score of 4). Nominal, ordinal, and interval data (i.e., plumage and behavioural/ecological characters) were scored more subjectively. For example, an “exceptional” character (e.g., a completely different colour in most of the plumage) received a score of 4, a “strong” character (e.g., a contrastingly different colour in most of the plumage) a score of 3, a “medium” character (e.g., a slightly different colour in a significant part of the plumage) a score of 2, and a “weak” character (e.g., a different shade in part of the plumage) a score of 1. Differences in behavioural/ecological characters were assessed using the available literature on *P*. *georgicus* and could only receive a score of 1. When the sum of two biometric, three plumage, and one behavioural/ecological character scores exceeded a total of 7, species status was warranted [46].

All analyses were conducted in Program R 3.3.1 [55] using the *effsize* [56] package. PCAs were visualised using the *ggplot2* [57] and *ggfortify* [58] packages.

### Taxonomy

The taxonomy of Procellariiformes remains in flux (e.g., [8, 9, 10]). We adhere here to the taxonomy of Gill *et al*. [18] in which *P*. *georgicus* is considered monotypic and a member of the Pelecanoididae family (order: Procellariiformes).

### Nomenclatural acts

The electronic edition of this article conforms to the requirements of the amended International Code of Zoological Nomenclature [59], and hence the new name contained herein is available under that Code from the electronic edition of this article. This published work and the nomenclatural act it contains have been registered in ZooBank, the online registration system for the ICZN. The ZooBank LSIDs (Life Science Identifiers) can be resolved and the associated information viewed through any standard web browser by appending the LSID to the prefix “http://zoobank.org/”. The LSID for this publication is: urn:lsid:zoobank.org:pub:F5A8048D-4B13-426A-AE7B-333BC400F327. The electronic edition of this work was published in a journal with an ISSN, and has been archived and is available from the following digital repositories: PubMed Central and LOCKSS.

### Ethical statement

All methods involving live birds in New Zealand were approved by institutional animal ethics committees (VUW AEC 22252 and VUW AEC 23283) and the New Zealand Department of Conservation (45407-FAU and 45907-FAU). Access to Codfish Island was granted by the New Zealand Department of Conservation (47920-LND and 52029-LND) and the Whenua Hou Committee.

All methods involving live birds on the Kerguelen Islands were approved by the ethics committee of the French Polar institute (Institut Paul-Emile Victor). Access to the Kerguelen Islands was granted by the Reserve Nationale des Terres Australes et Antarctiques Françaises and the Committee for Environmental Protection.

## Results

### Biometric characters

**Fig 4:**
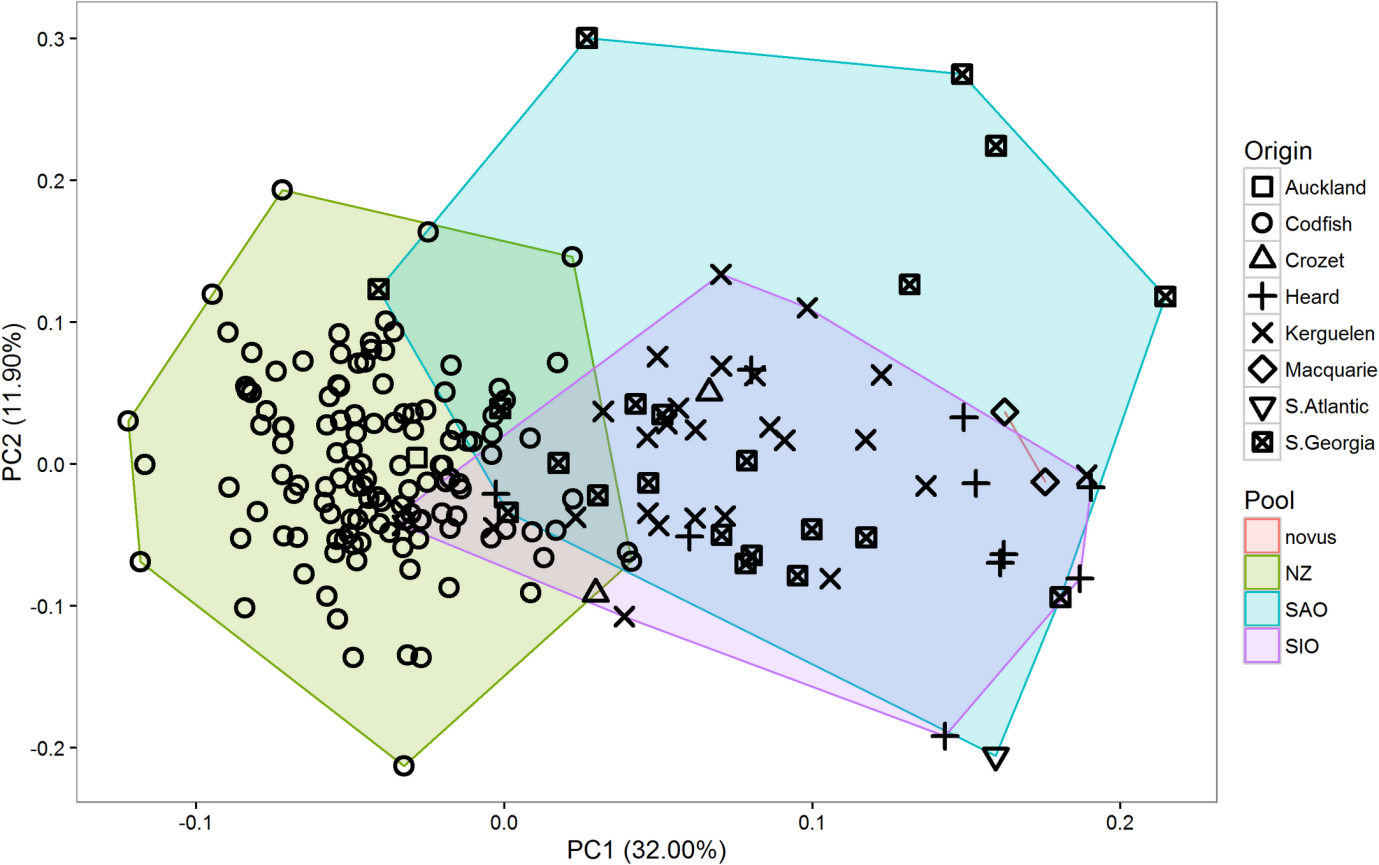
Principal component analysis (PCA) scatterplot of biometric characters of *Pelecanoides georgicus* samples. novus = *P*. *g*. *novus*, NZ = New Zealand, SAO = South Atlantic Ocean, SIO = South Indian Ocean. *n* = 190.

Results from the PCA of biometric characters showed almost complete overlap between the SAO and SIO populations, but only limited overlap between the SAO and NZ populations and the SIO and NZ populations (Table 2 and Fig 4). *P*. *g*. *novus* clustered with SAO and SIO populations. Results from multivariate factorial ANOVAs illustrated that significant differences among pools in biometric characters exist in PC1 (Table 2). Results from univariate factorial ANOVAs revealed differences in wing length (*f*2,192 = 12.146, *P* < 0.001), T6 length (*f*2,164 = 21.831, *P* < 0.001), bill length (*f*2,183 = 6.403, *P* = 0.002), bill depth (*f*2,50 = 12.685, *P* < 0.001), head length (*f*2,182 = 22.514, *P* < 0.001) and tarsus length (*f*2,183 = 14.143, *P* < 0.001). Results of pairwise comparisons using Tukey HSD tests revealed two groups within *P*. *georgicus* that could be readily distinguished by biometric characters: 1) consisting of the SAO and SIO pools and 2) the NZ pool (Table 3). The latter was overall larger.

**Table 2:** Principal component analysis (PCA) loadings of biometric characters of *Pelecanoides georgicus* samples.

Variable	PC1	PC2
Wing length	-0.377	-0.064
Length of T6	-0.391	0.257
Length of T1	-0.465	0.011
Bill length	0.081	0.495
Bill width	-0.157	0.630
Bill depth	-0.460	-0.007
Arch length	0.030	0.447
Head length	-0.343	-0.069
Tarsus length	-0.349	-0.262
Position of paraseptal process	0.073	0.121
Variance explained	32.00%	11.90%
*F* (ANOVA)	123.150	1.061
*df* (ANOVA)	186	186
*p* (ANOVA)	< 0.001	0.367

**Table 3:** Biometric characters of *Pelecanoides georgicus* populations.

Character	SAO	SIO	NZ	novus	SAO vs. SIO	SAO vs. NZ	SIO vs. NZ
Wing length	116.90 ± 0.82	117.82 ± 0.65	119.75 ± 0.23	111.00 ± 1.00		***	**
	(111–126; 29)	(109–126; 38)	(113–129; 111)	(110–112; **2**)		*d* = 0.95; 1	*d* = 0.65; 1
Length of T6	38.45 ± 0.77	37.41 ± 0.71	41.10 ± 0.21	36.50 ± 0.50		***	***
	(30–45; 22)	(32–42; 17)	(37–48; 128)	(36–37; **2**)		*d* = 1.03; 1	*d* = 1.52; 1
Length of T1	34.64 ± 0.52	34.19 ± 0.61	36.56 ± 0.71	30.50 ± 1.50			
	(29–39; 22)	(31–39; 16)	(35–39; 9)	(29–32; **2**)			
Tail fork depth	3.86 ± 0.80	3.06 ± 0.42	4.44 ± 0.60	6.00 ± 1.00			
	(-4-11; 22)	(0–7; 16)	(1–7; 9)	(5–7; **2**)			
Bill length	14.17 ± 0.18	14.16 ± 0.24	13.46 ± 0.11	14.65 ± 0.25			*
	(12.3–15.8; 22)	(11.4–17.4; 38)	(11.0–17.2; 126)	(14.4–14.9; **2**)			*d* = 0.54; 1
Bill width	8.29 ± 0.23	8.36 ± 0.08	8.52 ± 0.04	7.40 ± 0.00			
	(6.7–11.3; 22)	(7.5–9.7; 38)	(7.4–10.0; 126)	(7.4–7.4; **2**)			
Bill depth	7.66 ± 0.11	7.67 ± 0.14	8.71 ± 0.20	7.85 ± 0.15		***	***
	(6.9–8.7; 21)	(6.7–9.5; 22)	(7.8–9.4; 10)	(7.8–8.0; **2**)		*d* = 1.97; 1	*d* = 1.60; 1
Arch length	5.61 ± 0.17	5.07 ± 0.14	5.28 ± 0.09	6.20 ± 0.40			
	(4.6–6.9; 17)	(3.7–5.9; 21)	(3.3–7.2; 80)	(5.8–6.6; **2**)			
Head length	49.72 ± 0.39	49.50 ± 0.50	51.70 ± 0.14	50.20 ± 1.10		***	***
	(46.4–54.2; 21)	(36.7–56.5; 38)	(45.1–55.5; 126)	(49.1–51.3; **2**)		*d* = 1.23; 1	*d* = 1.08; 1
Tarsus length	24.16 ± 0.46	24.37 ± 0.16	25.38 ± 0.11	23.60 ± 1.10		***	***
	(19.3–27.2; 22)	(22.0–26.1; 38)	(22.0–28.6; 126)	(22.5–24.7; **2**)		*d* = 0.89; 1	*d* = 0.88; 1
Position of paraseptal process	53.33 ± 2.11	53.57 ± 1.69	53.23 ± 0.56	60.00 ± 10.00			
	(50–60; **6**)	(50–70; 14)	(40–70; 96)	(50–70; **2**)			
Maximum cumulative Tobias *et al*. (2010) score					0	2	2

Data presented are mean ± standard error of mean (minimum-maximum; *n*) in mm. Significance levels are indicated with asterisks (blank *P* > 0.05

* *P* < 0.05

** *P* < 0.01 and

*** *P* < 0.001

ANOVA, followed by Tukey HSD tests, unless *n* < 7 (bold)). Significance levels are followed by Cohen’s *d* effect sizes and Tobias *et al*. [46] scores, of which the two largest are summed. SAO = South Atlantic Ocean, SIO = South Indian Ocean, NZ = New Zealand, novus = *P*. *g*. *novus*.

### Plumage characters

**Fig 5:**
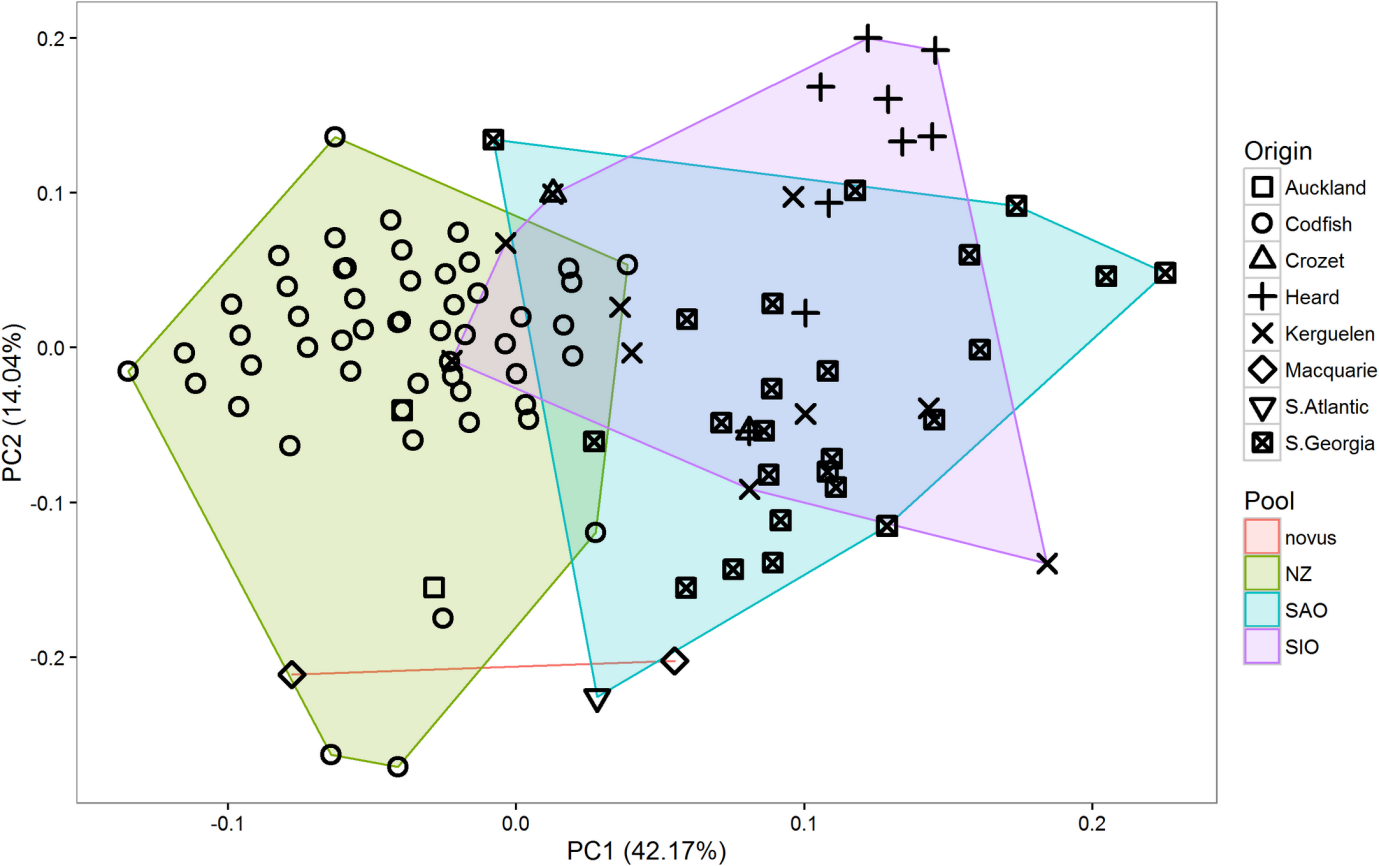
Principal component analysis (PCA) scatterplot of plumage characters of *Pelecanoides georgicus* samples. novus = *P*. *g*. *novus*, NZ = New Zealand, SAO = South Atlantic Ocean, SIO = South Indian Ocean. *n* = 169.

**Fig 6:**
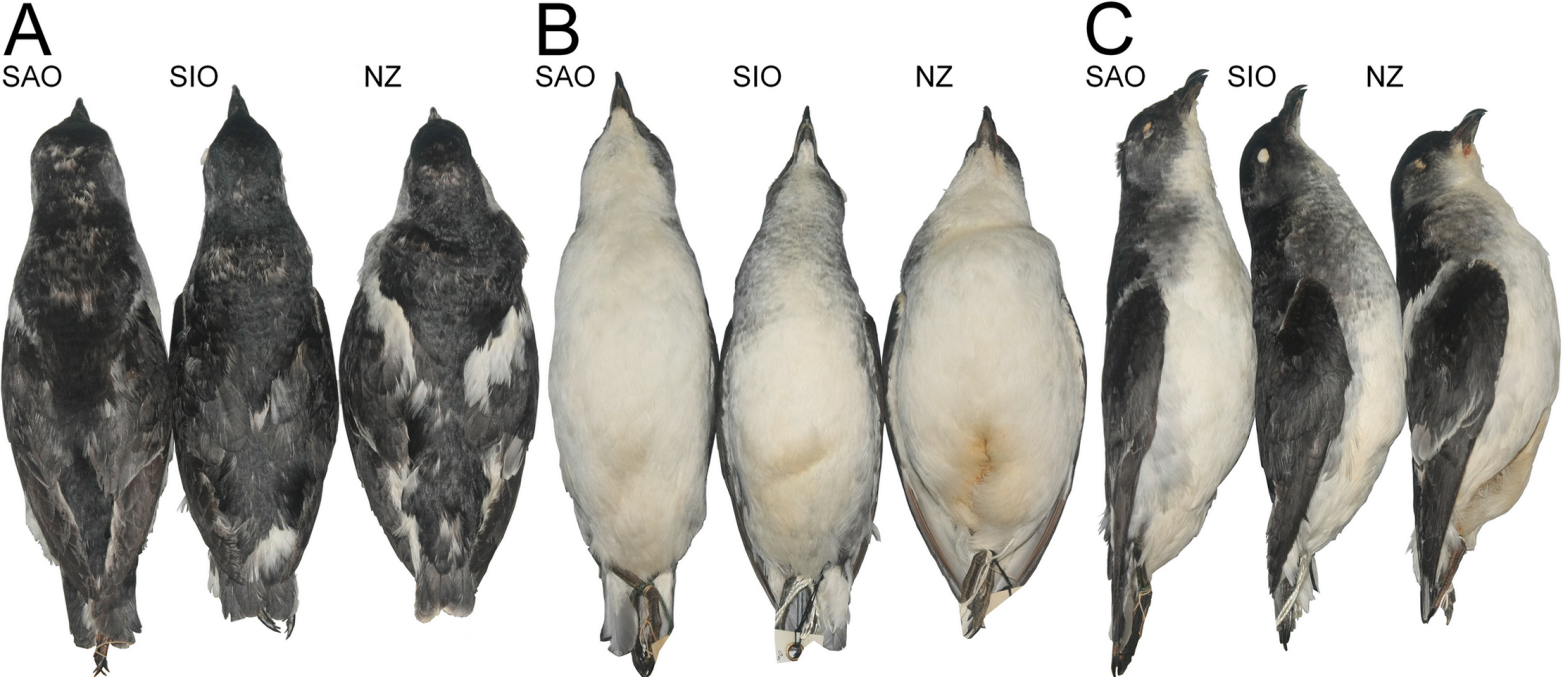
Study skins of *Pelecanoides georgicus* from different populations (Johannes H. Fischer). (A) Dorsal view. (B) Ventral view. (C) Lateral view. SAO = NMNZ OR.18421; origin: South Georgia, U.K., South Atlantic Ocean. SIO = NMNZ OR.24768; origin: Heard Island, Australia, South Indian Ocean. NZ = NMNZ OR.21631; origin: Dundas Island, Auckland Islands, New Zealand. Note differences in bill depth (NZ having the highest/deepest), collar extent (SIO having the largest), extent of contrasting scapulars (NZ having the largest), and contrasting white markings on secondaries (NZ having the largest) among others.

Results from the PCA of plumage characters revealed considerable overlap between the SAO and the SIO population, but very limited overlap between the SAO and NZ and the SIO and NZ populations (Table 4 and Fig 5). *P*. *g*. *novus* did not show as a clear separate cluster. Results from multivariate factorial ANOVAs illustrated that significant differences among pools in plumage characters existed in both PC1 and PC2 (Table 4). Results from univariate Kruskal-Wallis rank sum tests revealed differences in collar extent (*χ*^2^_2_ = 73.339, *P* < 0.001), extent of contrasting scapulars (*χ*^2^_2_ = 47.775, *P* < 0.001), extent of secondary markings (*χ*^2^_2_ = 67.647, *P* < 0.001), shape of secondary markings (*χ*^2^_2_ = 136.550, *P* < 0.001), ear-covert colour (*χ*^2^_2_ = 50.289, *P* < 0.001), collar colour (*χ*^2^_2_ = 59.505, *P* < 0.001) and flank colour (*χ*^2^_2_ = 12.314, *P* = 0.002). Results of pairwise comparisons with Welch’s two-sample t-tests showed that three groups (SAO, SIO, and NZ) could be distinguished from each other (Table 5 and Fig 6). Overall, the plumage of NZ birds was paler and more contrasting than that of birds from other populations.

**Table 4:** Principal component analysis (PCA) loadings of plumage characters of *Pelecanoides georgicus* samples.

Variable	PC1	PC2
Contrasting ear covert extent	0.250	-0.451
Collar extent	0.454	0.026
Contrasting scapular extent	-0.334	-0.370
Contrasting secondary marking extent	-0.384	-0.131
Contrasting secondary marking shape	-0.355	0.256
Contrasting ear covert colour	0.365	-0.071
Collar colour	0.392	-0.319
Flank colour	0.246	0.685
Variance explained	42.17%	14.04%
*F* (ANOVA)	132.840	15.095
*df* (ANOVA)	165	165
*p* (ANOVA)	< 0.001	< 0.001

**Table 5:** Plumage characters of *Pelecanoides georgicus* populations.

Character	SAO	SIO	NZ	novus	SAO vs. SIO	SAO vs. NZ	SIO vs. NZ
Contrasting ear covert extent (1–4)	3.46 ± 0.13	3.23 ± 0.15	3.18 ± 0.18	4.00 ± 0.00			
	(2–4; 29)	(2–4; 22)	(2–4; 11)	(4–4; **2**)			
Collar extent (1–4)	2.75 ± 0.14	3.27 ± 0.19	1.60 ± 0.05	2.00 ± 1.00	*	***	***
	(2–4; 29)	(2–4; 22)	(1–3; 112)	(1–3; **2**)	2	2	3
Contrasting scapular extent (1–4)	2.18 ± 0.13	2.00 ± 0.15	3.09 ± 0.07	3.00 ± 1.00		***	***
	(1–4; 29)	(1–4; 22)	(2–4; 116)	(2–4; **2**)		2	2
Contrasting secondary marking extent (1–5)	2.11 ± 0.08	2.14 ± 0.18	3.16 ± 0.07	3.00 ± 0.00		***	***
	(1–3; 29)	(1–5; 22)	(2–5; 115)	(3–3; **2)**		1	1
Contrasting secondary marking shape (1–4)	2.89 ± 0.10	3.81 ± 0.15	4.00 ± 0.00	4.00 ± 0.00	***	***	
	(1–4; 29)	(1–4; 21)	(4–4; 115)	(4–4; **2**)	1	1	
Contrasting ear covert colour (1–5)	3.43 ± 0.12	3.32 ± 0.10	2.57 ± 0.05	3.50 ± 0.50		***	***
	(2–4; 29)	(3–4; 22)	(2–4; 117)	(3–4; **2**)		1	1
Collar colour (1–5)	3.07 ± 0.08	3.05 ± 0.05	1.87 ± 0.08	2.00 ± 1.00		***	***
	(2–4; 29)	(3–4; 22)	(1–3; 112)	(1–3; **2**)		1	1
Flank colour (1–5)	2.10 ± 0.12	2.43 ± 0.11	1.64 ± 0.15	1.00 ± 0.00		*	***
	(1–3; 22)	(2–3; 21)	(1–2; 11)	(1–1; **2**)		1	1
Maximum cumulative Tobias *et al*. (2010) score					3	5	6

Data presented are mean ± standard error of mean (minimum-maximum; *n*). Significance levels are indicated with asterisks (blank *P* > 0.05

* *P* < 0.05

** *P* < 0.01 and

*** *P* < 0.001

Kruskal-Wallis rank sum tests followed by Welch’s two-sample t-tests, unless *n* < 7 (bold)). Significance levels are followed Tobias *et al*. [46] scores, of which the three largest are summed. SAO = South Atlantic Ocean, SIO = South Indian Ocean, NZ = New Zealand, novus = *P*. *g*. *novus*.

### Phenotypic species delimitation test

All Tobias *et al*. [46] scores corresponding with pairwise comparison for biometric and plumage characters are provided in Tables 3 and 5, respectively. The SAO population differed from the SIO population in only two plumage characters: collar extent (reasonably prominent in SAO, while extensive in SIO; score = 2) and the shape of contrasting secondary markings (limited to inner vane in SAO, while present on both inner and outer vane in SIO; score = 1). The total Tobias *et al*. [46] score when comparing SAO with SIO was thus 3.

The NZ population differed from the SAO population through five biometric and seven plumage characters, most prominent of which are: deeper bills (score = 1), longer heads (score = 1), collar extent (very limited in NZ, while reasonably prominent in SAO; score = 2), contrasting scapulars extent (very prominent in NZ, while limited in SAO; score = 2), and contrasting secondary marking extent (large and prominent in NZ, while limited in SAO; score = 1). In addition, one behavioural/ecological character was scored. The NZ population specialises in breeding in sandy foredunes at sea level [36], while the SIO and SAO populations breed in scree at high altitudes [19, 38]; score = 1). The total Tobias *et al*. [46] score when comparing NZ with SAO was thus 8.

The NZ population differed from the SIO population through six biometric and six plumage characters, most prominent of which are: a longer T6 (score = 1), deeper bills (score = 1), collar extent (very limited in NZ, while extensive in SIO; score = 3), contrasting scapulars extent (very prominent in NZ, while limited in SIO, score = 2), and contrasting secondary marking extent (large and prominent in NZ, while limited in SIO; score = 1). In addition, the difference in breeding habitat (sandy foredunes at sea level [36] vs. scree at higher altitudes [19, 38]; score = 1) was scored. The total Tobias *et al*. [46] score when comparing NZ with SIO was thus 9.

## Discussion

Our results show that the NZ *P*. *georgicus* population is distinct from all other populations and exhibits five biometric and six plumage characters that are (at least in combination) diagnostic. Results of the quantitative phenotypic species delimitation test [46] showed that the NZ population warrants species status as the threshold of 7 is surpassed in both key comparisons (NZ vs. SAO and NZ vs. SIO). In addition, results suggest that *P*. *g*. *novus* [25], is a junior synonym of the nominate form of *P*. *georgicus* [16] (the SIO and SAO populations), as currently widely recognised [18, 31, 32]. Furthermore, the SAO *P*. *georgicus* population is very similar to the SIO population with only limited differentiation in plumage characters.

Despite providing evidence for the distinctiveness of the NZ *P*. *georgicus* population, our analyses are not exhaustive, e.g., we have not included quantitative genetic analyses, bioacoustics, nor moulting strategies in our analyses. Results of a preliminary bioacoustic analysis indicate slight differences, at least between the NZ and the SIO and SAO populations (calls from birds from South Georgia and the Crozet Islands are audibly coarser than calls from birds from Codfish Island; [38, Fischer unpub. data]. We suspect that an extensive bioacoustic analysis will provide further insights on the relationships of the different populations. We lodged a sound recording from Codfish Island in a public library to facilitate such a comparison (www.xeno-canto.org; catalogue number XC295661). Furthermore, a detailed analysis of moulting strategies between the three populations could be informative, for different moulting strategies can also provide clues on the species status of other cryptic Procellariiform taxa [60]. Anecdotal data suggest some differences in moulting strategies between populations, as birds from New Zealand are still moulting primaries in late September in contrast with the other *P*. *georgicus* populations that moult between April and June [22]. While numerous species have been described based solely on phenotypic characters, even in recent years (e.g., [2, 61–63]), a thorough molecular analysis of *P*. *georgicus* would likely provide further useful insights. Such an analysis would complement preliminary results [26, 39]. Moreover, we encourage such investigations to extend beyond the *P*. *georgicus* complex and include the *P*. *urinatrix* complex or even all *Pelecanoides* species to investigate potential further cryptic species within this genus.

Our results indicate that the birds on Codfish Island, currently recognised as *P*. *georgicus*, are a distinct species, but both biometric and plumage characters overlap with other *P*. *georgicus* populations. All *Pelecanoides* species are very similar in both biometrics and plumage [19–23, 64] and consequently confusing to separate. Therefore, the documented range of overlap in biometric and plumage characters is not unexpected. However, the detailed analyses presented here indicate that the NZ population indeed warrants species status based on the criteria of diagnosability and degree of differentiation. Diagnosability was the most frequently applied species criterion in a review of species criteria in avian taxonomy studies [65]. The NZ population of *P*. *georgicus* exhibits eleven phenotypic characters that appear, at least in combination, diagnostic. Another commonly applied criterion was the degree of differences [65]. The results of the phenotypic species delimitation test [46] suggest that the NZ population of *P*. *georgicus* differs too extensively from other populations to be treated as subspecies. Given the common use of diagnosability and degree of difference as species criteria [65], the recent broad, international coverage of the applied phenotypic species delimitation test [46] in assessing species limits (e.g., [24, 31, 66, 67]), and previous molecular [26], osteological [39], and parasitological [40] work, we conclude that the NZ *P*. *georgicus* population merits species status. No name has previously been assigned to the New Zealand population of *P*. *georgicus* [18]. We therefore propose to name this species:

***Pelecanoides whenuahouensis sp*. *nov*.** urn:lsid:zoobank.org:act:9EF2466F-9AE7-40F4-A0D0-D9822566C4F5

### Holotype

NMNZ OR.21058 (adult female), collected at Codfish Island, New Zealand, on 22 September 1978 by MJ Imber (S1 Fig). This study skin was previously assigned to *P*. *georgicus* [16].

### Paratypes

The following 11 study skins were all previously assigned to *P*. *georgicus* [16] and were used in the species description of *P*. *whenuahouensis*: NMNZ OR.21631 (adult female; Fig 6), from Dundas Island, Auckland Islands, New Zealand, and BMNH.1842.12.16.41 (sex and age unknown), from Enderby Island, Auckland Islands, New Zealand, both collected in November/December 1840 by the James Clark Ross Antarctic expedition of the Office of the Admiralty and Marine Affairs. NMNZ OR.21057 (adult male) collected at Codfish Island, New Zealand, on 22 September 1978 by MJ Imber. NMNZ OR.21070 (female) and NMNZ OR.21071 (adult female), both collected at Codfish Island, New Zealand, on 17 November 1978 by PC Harper. NMNZ OR.27537 (adult male), NMNZ OR.27538 (adult male), NMNZ OR.27539 (adult female), NMNZ OR.27540 (adult female) and NMNZ OR.27541 (adult male), all collected at Codfish Island, New Zealand, in September/October 2003 by the New Zealand Department of Conservation.

### Etymology

*P*. *whenuahouensis* is named after the name of Codfish Island in the Māori language/*Te Reo Māori*: *Whenua Hou* (pronounced 'fɛnua 'hou, meaning ‘new land’ [68]). This island hosts the only extant colony of this species [30, 36]. This name was selected by the *Ngāi Tahu*, the Māori people who still hold a genealogical, cultural, and spiritual connection to both the island and this species, which they consider a *taonga* (treasure).

### Common name

We propose the English common name ‘Whenua Hou Diving Petrel’.

### Generic placement

*P*. *whenuahouensis* clearly belongs in *Pelecanoides*, [69], (family: Pelecanoididae, order: Procellariiformes) based on a combination of black and white plumage, short, paddle-like wings, short tail, small and compact build, and bill morphology (short, broad based bill with hooked tip, a paraseptal process in nostrils, and gular pouch) [18, 22, 23].

### Diagnosis

*P*. *whenuahouensis* differs from *P*. *garnottii* [14], through bill morphology/coloration (a shorter, slimmer bill, with much smaller nostrils, the presence of lavender blue on the lower mandible, and a less well-defined paraseptal process (but both species have the paraseptal process placed at approximately 50%) and a smaller overall size (resulting in shorter wings, tarsi, and a much lower bodyweight) [21, 22, 23, 37]. *P*. *whenuahouensis*, however, does appear to have a longer tail than *P*. *garnottii* [23, 37]. Furthermore, *P*. *whenuahouensis* exhibits 1) a much larger extent of contrasting ear coverts, 2) continuous and pure white scapulars, 3) a limited (light grey) collar, 4) much paler (light grey) flanks and axillaries, and 5) white underwings including primaries [20, 22, 23, 70]. In addition, *P*. *whenuahouensis* can also be readily distinguished from *P*. *garnottii* based on vocalisations [21].

*P*. *whenuahouensis* differs from *P*. *magellani*, [15], through bill morphology/coloration (a shorter, but wider and deeper bill, with more lavender blue on the lower mandible, and the placement of the paraseptal process at approximately 50%) and a smaller overall size (resulting in shorter wings and tarsi) [21, 22, 23, 37, 71]. *P*. *whenuahouensis* also exhibits a less contrasting and a less mottled plumage than *P*. *magellani*. Specifically, *P*. *whenuahouensis* 1) exhibits darker (light grey instead of white) and less well-defined ear coverts, 2) lacks the white tips on the back and rump feathers, upperwing coverts, and tertails, 3) shows continuous (instead of mottled) contrasting, white scapulars, 4) exhibits a much smaller and light grey collar, and 5) has much paler (light grey) flanks and axillaries [20, 21, 22, 23, 70, 71]. Vocalisations of *P*. *magellani* remain unknown [21, 71].

*P*. *whenuahouensis* differs from most *P*. *urinatrix* [17], subspecies through bill morphology/coloration (a slightly shorter, but wider bill, with a shorter mandible arch length, convergent bill sides, the paraseptal process placed at approximately 50%, and a larger amount of lavender blue on the lower mandible), having a longer tail with a distinct tail fork, and generally shorter tarsi [21, 22, 23, 37]. Furthermore, when compared to most *P*. *urinatrix* subspecies, *P*. *whenuahouensis* exhibits, 1) generally well-defined, paler (light grey) ear covers, 2) the complete absence of any grey mottling on the throat, 3) well-defined contrasting, white scapulars, consisting of completely white feathers, 4) extensive, contrasting white markings on the secondaries, 5) white underwings, including white inner vanes of the outermost primaries (P10), 6) a very limited, light grey collar, 7) lighter (light grey) flanks and axillaries [20–23, 38, 70, 71]. It should be noted that *P*. *u*. *chathamensis* [16] breeds in low numbers alongside *P*. *whenuahouensis* in the Codfish Island dunes and all criteria listed above allow easy separation between the two species in the hand [36, 44]. *P*. *u*. *exsul* [42], which used to breed alongside *P*. *whenuahouensis* on the Auckland Islands before *P*. *whenuahouensis* was extirpated [23, 30], however, is harder to distinguish from *P*. *whenuahouensis*, owing to the similarity in bill morphology (e.g., convergent bill sides; [23]) and the plumage variation shown within *P*. *u*. *exsul* [19, 37, 38]. Hence, the differentiation between *P*. *whenuahouensis* and *P*. *u*. *exsul* relies on 1) assessment of paraseptal process placement, 2) tail shape, and 3) outer primary coloration [23, 30, 38]. However, darker individuals of *P*. *u*. *exsul* are easier to identify based on their grey underwings, an extensive grey collar, and extensive grey mottling on the throat ([19, 23, 30, 37]. Moreover, *P*. *whenuahouensis* has a continuous black line on the hind tarsus in all plumages, while most populations of *P*. *urinatrix* only exhibit this feature in juveniles [19, 38, 71]. *P*. *whenuahouensis c*an also be readily distinguished from *P*. *urinatrix* using vocalisations [19, 30, 36, 38, 41, 71].

*P*. *whenuahouensis* differs from all *P*. *georgicus* [16], populations by having 1) a deeper bill, 2) a longer head, 3) longer wings, 4) longer outermost tail feathers, and 5) longer tarsi. *P*. *whenuahouensis* also differs from *P*. *georgicus* by having a more contrasting plumage. Specifically, *P*. *whenuahouensis* exhibits 1) light grey ear coverts, 2) well defined and prominent, contrasting white scapulars, 3) large contrasting white markings on secondaries, 4) a very limited collar that is only visible on the breast sides, 5) the light grey coloration of the limited collar, and 6) light grey flanks (Tables 3 and 5, Fig 6). Furthermore, the in *P*. *whenuahouensis* the claw on the inner toe does not extend beyond the base of the claw on the middle toe as it does in *P*. *georgicus* [23].

### Description of the holotype

HEAD: the forehead is dark brown, while the crown is glossy black. The nape is also glossy black, but feather bases are light grey. The lores are dark brown. The cheeks and ear coverts are mottled and light grey, creating a prominent and contrasting pattern. (S1 Fig).

UPPERPARTS: The mantle feathers have light grey bases and glossy black tips. The scapulars are pure white, prominent, contrasting, and almost connected above the rump. The rump and back are glossy black, but feather bases are white. The uppertail coverts have broad white bases and glossy black tips (S1 Fig).

UNDERPARTS: The chin, throat and upper breast, lower breast, belly and undertail coverts are dirty white. The breast and neck sides are mottled light grey. The flanks are smudged light grey (S1 Fig).

WING: The upperwing coverts (both primary and secondary) and tertials are glossy black with a brown hue and glossy black tips. Dorsally, the primaries are dull black with a dark brown hue. Ventrally, the primaries have dirty white inner vanes and light grey outer vanes. The outermost functional primaries (P10) are the longest. The secondaries are dull black on the outer vane dorsally and dark grey ventrally, while the inner vane is light grey (both dorsally and ventrally). The secondaries have broad white tips extending towards the base on both inner and outer vanes. The secondary feather tips are fringed outwards. The underwing coverts (both primary and secondary) are pure white (S1 Fig).

TAIL: The rectrices are dull black dorsally and dark grey ventrally, apart from the outermost pair (T6), which is grey on the inner vane. The two outer rectrices (T6 and T5) are longer than the inner rectrices (T4-T1; T1 being the shortest), resulting in a shallow, but well defined, tail fork.

BARE PARTS: the bill is black, with a hooked tip, a broad base and convergent bill sides. The lower mandible arch sides are dull brown, suggesting a faded colour (live birds have lavender blue lower mandible arch sides). The nostrils (nasal tubes) are black, parallel and facing upwards with a medial paraseptal process. The gular pouch is dark grey, suggesting fading (live birds have pale blue patterns on the gular pouch). The legs and webbed feet are dull brown, suggesting a faded colour (live birds have cobalt blue legs), with a hint of a black line on the back of the tarsus (live birds have a continuous black line on the hind tarsus). The claws are black and slightly flattened.

### Variation in type series

NMNZ OR.21631 (adult female; Fig 6) differs from holotype by having: 1) a dark grey brow, 2) a slightly larger extent of contrasting white scapulars, and 3) a small, grey collar. NMNZ OR.21070 (female) differs from holotype by having: 1) black cheeks, 2) grey ear coverts covering a slightly smaller area, 3) a slightly smaller contrasting ear patch extent, 4) a slightly smaller extent of contrasting white scapulars, and 5) a light grey outermost pair of rectrices (T6). NMNZ OR.21071 (adult female) differs from holotype by having: 1) grey ear coverts, 2) a slightly larger extent of contrasting white scapulars, 3) a small, grey collar, and 4) a light grey outermost pair of rectrices (T6). NMNZ OR.27539 (adult female) differs from holotype by having: 1) a dark grey brow, 2) grey ear coverts, 3) black cheeks, 4) a slightly larger extent of contrasting white scapulars, 5) a small, grey collar, 6) a light grey outermost pair of rectrices (T6), and 7) pure white flanks. NMNZ OR.27540 (adult female) differs from the holotype by having: 1) black cheeks, 2) grey ear coverts, 3) a slightly larger extent of contrasting white scapulars, 4) a small, grey collar, 5) pure white flanks, and 6) slightly small extent of contrasting white markings on secondaries.

NMNZ OR.21057 (adult male) differs from holotype by having: 1) grey ear coverts and 2) a light grey outermost pair of rectrices (T6). NMNZ OR.27537 (adult male) differs from holotype by having: 1) black cheeks, 2) grey ear coverts, 3) a slightly larger extent of contrasting white scapulars, 4) a small, grey collar, and 5) a light grey outermost pair of rectrices (T6). NMNZ OR.27538 (adult male) differs from holotype by having: 1) a dark grey brow, 2) black cheeks, 3) a slightly larger extent of contrasting ear coverts, 4) a slightly larger extent of contrasting white scapulars, 5) a small, grey collar, 6) pure white flanks, and 7) a light grey outermost pair of rectrices (T6). NMNZ OR.27541 (adult male) differs from holotype by having: 1) black cheeks, 2) grey ear coverts, 3) a slightly larger extent of contrasting ear coverts, and 4) a small, grey collar.

BMNH.1842.12.16.41 (sex unknown) differs from holotype by having: 1) grey ear coverts, 2) a small grey collar, and 3) pure white flanks.

### Identification at sea

Diving Petrels are notoriously difficult to identify at sea [22] and even in the hand [23, 38]. For example, distinguishing *P*. *urinatrix* from *P*. *georgicus* at sea is virtually impossible [19, 21, 22, 71]. Even high resolution at sea photographs are unlikely to clearly depict the subtle differences between *P*. *georgicus* and *P*. *whenuahouensis*. Therefore, at sea sightings are unlikely to be able to elucidate the pelagic range of *P*. *whenuahouensis*. However, *Pelecanoides* spp. with pure white underwings seen in inshore waters west of Stewart Island likely pertain to *P*. *whenuahouensis* as the occurrence of *P*. *georgicus* in these waters is highly unlikely.

### Distribution

All known study skins of *P*. *whenuahouensis* originate from either Dundas Island, Enderby Island (both Auckland Islands, New Zealand), or Codfish Island, New Zealand (Fig 1). *P*. *whenuahouensis* remains extant only on Codfish Island, where it breeds in a minute (0.018 km^2^) strip of coastal, sandy foredunes in Sealers Bay [30, 36, 44]. The historic distribution of *P*. *whenuahouensis* in New Zealand likely encompassed the Otago Peninsula on the South Island, Mason’s Bay on Stewart Island, Enderby and Dundas Islands on the Auckland Islands and the Chatham Islands [6, 29, 30, 40, 72].

The offshore distribution of *P*. *whenuahouensis* remains unknown. Prey species found in two specimens indicate that *P*. *whenuahouensis* forages on the edge of the continental shelf during the breeding season [43]. The only documented *P*. *georgicus* record for Australia (Bellambi Beach, New South Wales) likely pertained to *P*. *whenuahouensis*, based on the reported biometrics (most notably a tail length of 41 mm; [73]), indicating at least considerable vagrancy potential, and perhaps a larger offshore distribution than previously assumed [19], as recently demonstrated in *P*. *u*. *urinatrix* [74].

### Breeding habitat

*P*. *whenuahouensis* breeds in burrows in coastal sand dunes. It exhibits an extreme preference for foredunes (0–20 m from spring tide line) with steep, seaward-facing slopes, high sand flux and 50–80% plant cover [36]. *P*. *whenuahouensis* appears to be tolerant of the current suite of invasive plants (most prominently *Dactylus glomerata*, *Holcus lanatus*, and *Hypochaeris radicata*) at Codfish Island [75].

### Breeding biology

Information on the breeding biology of *P*. *whenuahouensis* remains anecdotal [44, 75–80]. *P*. *whenuahouensis* presumably returns to Codfish Island from its unknown wintering grounds in early September. Eggs hatch in late November, but exact incubation times are unknown. Chicks fledge in early to mid-January after approximately 50 days. Both parents care for eggs and chicks. Nocturnal change-over rates of adults are approximately four days during incubation and one day during chick rearing [76, 80]. Adults appear to leave the colony 1–4 days before chicks fledge.

## Feeding ecology

Insights on prey items of *P*. *whenuahouensis* during the breeding and non-breeding season remain equally anecdotal. All *Pelecanoides* species are wing-propelled pursuit-divers and largely planktivorous [23, 81]. Two specimens of *P*. *whenuahouensis* collected during the breeding season had eusphausiids, small fish, and small squids in their stomachs [43].

### Conservation implications

*P*. *georgicus* is currently listed as ‘Least Concern’ by the IUCN [24]. Our proposed split of *P*. *georgicus* would not change the conservation status for the SAO and SIO *P*. *georgicus* populations. Both still number in the millions, both have a large range, and there are no indications of any current population declines [19, 21]. The status of the *P*. *georgicus* population on Macquarie Island and surrounding islets (which is the only *P*. *georgicus* population in the Southern Pacific Ocean), however, is precarious as the known population remains extremely low [27, 28]. On the other hand, the species appears to have recolonized Macquarie Island following the eradication of introduced mammals [27].

In contrast, *P*. *whenuahouensis* is at an extremely high risk of extinction. The range of *P*. *whenuahouensis* has decreased dramatically since human colonisation of New Zealand. The species has been extirpated throughout most of its range, most likely due to introduced predators [6, 29, 40, 72] and *P*. *whenuahouensis* is now restricted to Codfish Island. While Codfish Island is now free of introduced predators [68], the population size of *P*. *whenuahouensis* remains minute [29]. The New Zealand Department of Conservation therefore, already considers this taxon ‘Nationally Critical’ in New Zealand [45]. Consequently, we propose that *P*. *whenuahouensis* to be listed as ‘Critically Endangered’ on the IUCN Red List. When applying the IUCN [82] criteria to *P*. *whenuahouensis*, it qualifies for listing as ‘Critically Endangered’, based on criteria B2ab (ii, iii) and C2a (ii). *P*. *whenuahouensis* has an extremely limited area of occupancy (0.018 km^2^) during the breeding season at only a single location [36]. Its habitat is degrading due to catastrophes such as storms and storm surges, which reduce the area of occupancy [36, 76, 77]. Furthermore, the estimated population size is extremely small (estimated at 150 adults in 2005 [29]), all individuals are part of this one population, and a decline is expected due to the impact of climate change, storms and storm surges during breeding seasons [36, 77]. Moreover, competition with *P*. *u*. *chathamensis* for nest sites may be a minor threat to *P*. *whenuahouensis* [44]. No current pelagic threats to *P*. *whenuahouensis* have been identified [80], but *P*. *georgicus* and other smaller Procellariiformes suffer from deck strikes on ships [83]. ‘Critically Endangered’ is indicative of an extremely high risk of extinction [82], and thus underlines the need for conservation prioritization for *P*. *whenuahouensis*.

To secure *P*. *whenuahouensis*, translocations within its historic range may be appropriate to establish a new colony and thus render the species less vulnerable to stochastic events [36, 84, 85]. However, before such a strategy can be considered, detailed information on breeding biology [44, 84, 85, 86] and population dynamics [87] of *P*. *whenuahouensis* will be required. Additionally, competition control, such as burrow flaps [88]), may be required if the *P*. *u*. *chathamensis* population within the *P*. *whenuahouensis* colony expands [44]. This technique has successfully reduced competition pressure between more aggressive, common Procellariiformes and less aggressive, threatened species on other islands [88]. Further research aimed at understanding the dynamics between the two species appears necessary. Finally, investigating the offshore distribution and the corresponding risk factors is required to appreciate all threats faced by *P*. *whenuahouensis*.

## Conclusion

Here, we provide evidence of the distinctiveness of the Whenua Hou Diving Petrel (*Pelecanoides whenuahouensis sp*. *nov*.; previously part of the South Georgian Diving Petrel *P*. *georgicus* [16] complex), which is a ‘Critically Endangered’ species. The conservation status of this species has remained “hidden” to global conservation interests due to its inclusion in a polytypic “species”. New Zealand, however, maintains a national threat classification system [45] and therefore, the dire situation of *P*. *whenuahouensis* has been acknowledged within New Zealand. Consequently, we advocate the continuing use of national threat classification systems, as in cases like this, it has complemented the global threat classification system, by protecting taxa for which the taxonomy is still unclear. In addition, we urge taxonomists to focus new research on polytypic species that are likely to include threatened taxa [2], for conservation efforts depend on species being a clear and single ecological unit.

## Supporting information

S1 File Abstract in the Māori language/*Te Reo Māori*. (PDF)

S2 File Video illustrating the exact biometric measuring techniques employed in this study. Accessible through https://youtu.be/gyJnRYW0NKY.
(MP4)

S1 Fig Lateral view of the holotype of *P*. *whenuahouensis* (NMNZ OR.21058) (Johannes H. Fischer). (TIF)
