# Supplementary material for: Analyses of phenotypic differentiations among South Georgian Diving Petrel (Pelecanoides georgicus) populations reveal an undescribed and highly endangered species from New Zealand
Source: PLoS One. 2018 Jun 27;13(6):e0197766. doi: 10.1371/journal.pone.0197766 (PMC6021066; doi:10.1371/journal.pone.0197766)
Supplement: S1 File — (PDF) [file pone.0197766.s001.pdf]

Nā ngā tātaritanga o ngā rerekētanga ā-āhua i waenga i ngā taupori Kōrure Rukukare (South Georgian Diving Petrel) (*Pelecanoides georgicus*) ka kitea he momo motuhake, kāore anō kia kōrerotia ki te ao, he tino mōrearea hoki te noho, nō Aotearoa taketake

He uaua te whakarōpū ingoa mō ngā momo noho mōrearea mā te hunga tiaki taiao, nā te mea, kei te whakawhirinaki te hunga mahi ki ngā whakarōpūtanga ingoa motuhake mō tēnā momo, mō tēnā momo. Nā ngā rerekētanga o ngā ripoinga whakaputa uri me ngā hua o tētahi tātaritanga rāpoi ngota tuatahi i tohu he momo motuhake, kāore anō kia whakamāramatia, te hapori o te kōrure rukukare (*Pelecanoides georgicus*) o Aotearoa. I inea e mātou ētahi pūāhua koiora 11, ā, i tohua e mātou ngā tauanga mō ētahi pūāhua huruhuru e waru i ētahi manu ora 143, me ētahi kiri manu 64, mai i te nuinga o ngā hapori o *P. georgicus*, hei aromatawai i ngā huānga ā-whakarōpūtanga ingoa. I tātaritia e mātou ngā rerekētanga ki ngā tātaritanga wae matua (PCA), ngā ANOVA huarea, me ngā whakamātautau whakahiato mātāmuatanga Kruskal-Wallis. E ai ki ngā hua, ko ngā manu takitahi o Aotearoa he rerekē tonu i ētahi atu *P. georgicus* i ētahi katoa atu o ngā hapori, inā rā ngā rerekētanga: 1) he roa kē atu ngā parirau, 2) he roa kē atu ngā hikumārō, 3) he whānui kē atu ngā ngutu, 4) he roa kē atu ngā pane, 5) he roa kē atu ngā wae, 6) he whāiti iho ngā tāhei o te kakī, 7) he whānui kē atu te horanga o ngā takenga parirau, 8) he nui kē atu ngā āpure kōhae i ngā huruhuru tuarua o te parirau, 9) he kōtea kē ake ngā pare taringa, 10) he kōtea kē ake ngā tāhei, ā, 11) he kōtea kē ake ngā taha. Waihoki, i whakamahia e mātou tētahi whakamātautau wehewehe momo ki ngā paearu tautohu i te maha o ngā rerekētanga ā-āhua; ā, e ai ki ngā hua kua kitea, he tika tonu kia kīia he momo motuhake te hapori o *P. georgicus* o Aotearoa. Koia mātou ka tapa atu nei i te ingoa *Pelecanoides whenuahouensis sp. nov*. ki tēnei momo hōu. Nā te whakahekenga taumaha i ōna ripoinga me te ruarua noa iho o ngā manu e toe ana i te ao nei (~150 ngā manu takitahi i tētahi huihuinga whakaputa uri kotahi i Whenua Hou/Codfish Island), he tika tonu kia uru tēnei momo ki te rārangi ‘Tino Mōrearea Rawa Atu’.
