# Supplementary figures and images for: Analyses of phenotypic differentiations among South Georgian Diving Petrel (Pelecanoides georgicus) populations reveal an undescribed and highly endangered species from New Zealand
Source: PLoS One. 2018 Jun 27;13(6):e0197766. doi: 10.1371/journal.pone.0197766 (PMC6021066; doi:10.1371/journal.pone.0197766)

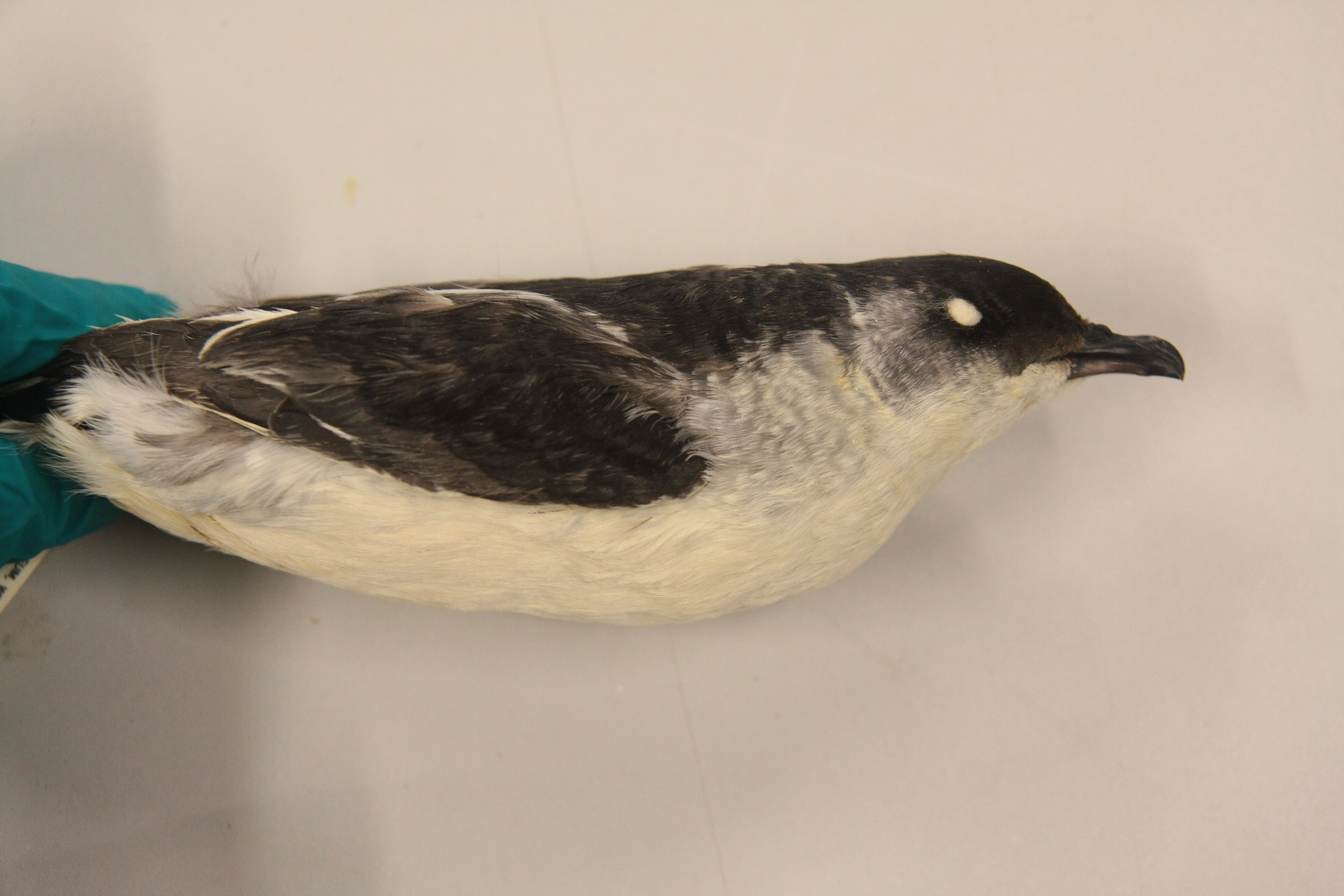

Supplement: S1 Fig — (TIF) [file pone.0197766.s003.tif]
